# Clinical case-series report of traumatic cauda equina herniation: A pathological phenomena occurring with thoracolumbar and lumbar burst fractures

**DOI:** 10.1097/MD.0000000000006446

**Published:** 2017-04-07

**Authors:** Liang Yan, Yang Liu, Baorong He, Jijun Liu, Zhenguo Luo, Dingjun Hao

**Affiliations:** a Department of Spinal Surgery; b Department of Orthopaedics; c Department of Anesthesiology, Hong Hui Hospital, Xi’an Jiaotong University College of Medicine, Shaanxi, China.

**Keywords:** burst fracture, cauda equina herniation, thoracolumbar, traumatic

## Abstract

**Rationale:**

Burst fractures in thoracolumbar and lumbar spine typically occur from severe trauma, which may result in cauda equina herniation (CEH). In this study, we attempted to document the incidence and evaluate the sequelae of CEH that were found during decompression and fusion surgery for patients with burst fractures.

**Patient concerns:**

A total of 416 patients were enrolled in this study.

**Diagnoses:**

The patients had been operated on through an anterior or posterior approach for treatments of thoracolumbar and lumbar burst fractures at our department between June 1, 2008 and June 1, 2011.

**Interventions:**

A retrospective analysis of hospital records and a review on radiographs were performed. Data regarding demographics, injury mechanism, radiographs, surgical procedures, outcomes and follow-ups were collected and analyzed.

**Outcomes:**

The CEH was observed in 49 patients (12%), including 40 males and 9 females with a mean age of 33.7 years old. The posterior approach group included 301 patients (215 males and 86 females), with 13% incident rate for CEH (40/301). The anterior approach group included 115 patients (80 males and 35 females), with 8% incident rate for CEH (9/115). Forty-four patients (90%) with CEH had neurological deficits; while other 5 patients (10%) were neurologically intact but entrapments of a significant proportion of their cauda equina rootlets in the dorsal lamina fracture were observed during operations. Both vertebrae burst fracture and lamina fracture were observed in 95% patients with posterior CEH (38 out of 40).

**Lessons:**

Traumatic CEH were found in 12% of the patients with thoracolumbar and lumbar burst fractures that were treated by surgery. Patients with a lumbar burst fracture showing neurological deficits in combination with a laminar fracture have an increased risk of traumatic CEH.

## 1 Introduction

Spinal “burst fracture” was first described by Holdsworth^[1]^ and redefined by Denis in 1983.^[2]^ Burst fractures are a type of compression fracture with failure in the middle and anterior spinal columns due to a high-energy axial loading, which account for 14% of all spinal injuries. The most common sites of injury caused by burst fractures are thoracolumbar and lumbar spine. The mechanism of injury is usually associated with severe trauma that is resulted from falls and traffic accidents. The management of thoracolumbar and lumbar burst fractures still remains a number of important treatment challenges. Both conservative management and surgery are among the options of treatment, but controversies present regarding to the indications for nonoperative or operative management of these factures. Burst fractures without neurological deficits are usually managed by conservative approaches. However, it may lead to ongoing neurogenic pain, movement deficits, and progressive spinal deformity. While surgical treatment usually involves instrumented intervertebral fusion, with or without spinal decompression. It has the advantage of restoration of vertebral height, correction of kyphosis, decompression of neurostructures, and maintenance of stability. There are 2 major surgical approaches, posterior and anterior surgeries.^[1–3]^Anterior approach renders a direct visualization of ventral canal and the greatest degree of canal decompression. However, it increases time and risk of surgery due to implicating chest and abdominal organs as well as major blood vessels. Posterior surgery is an effective approach for most burst fracture with satisfactory clinical outcomes. It can detect and decompress the posterolateral of spinal cord. However, inadequate decompression and implantation failure are among the main limitations for this approach. Dural laceration and cauda equina herniation (CEH) caused by burst fractures can be detected through an either anterior or posterior approach. Few reports have been published regarding CEH resulting from spinal burst fractures.^[3–6]^ The aim of this retrospective study was to document the incident rate of burst fractures-caused CEH and evaluate the sequelae.

## 2 Methods

### 2.1 Subjects

This retrospective study was approved by the Ethical Committee of Hong Hui Hospital, Xi’an Jiaotong University College of Medicine. All the patients had signed the informed consent form to allow their information to be used for the research purposes. A total of 416 patients were enrolled in this study. A retrospective analysis of hospital records and a review on radiographs were performed. All the subjects had been treated for thoracolumbar and lumbar burst fractures at our department between June 1, 2008 and June 1, 2011. The subjects had been operated on through an anterior or posterior approach between day 1 and 12 after injury (mean, 4 days). There were 295 male and 121 female patients, with an average age of 38.6 years old (range, 15–64 years old). Before surgery, neurological status regarding motor, sensory, control of the bowel and bladder was evaluated, recorded, and rated by Frankel's classification. Each patient had an anteroposterior and lateral plain radiograph and computed tomography scans of the fracture site, and when available, magnetic resonance imaging as well. All patients were kept on strict bed rest and those with neurological compromise were given parenteral high-dose steroids and histamine blockers.

### 2.2 Surgical technique

#### 2.2.1 Posterior approach

Posterior approach is the main treatment method used for thoracolumbar and lumbar burst fractures which renders open reduction and internal fixation using pedicle screw. Patients were placed in a prone position, followed by general anesthesia. C-arm images were used to locate the pedicle of the involved segment, followed by a midline incision centered over it. The dissection was carried out by layers till the tip of the spinous processes exposed. Subperiosteally the paraspinal muscles were detached to the outer edge of the facet to expose the bilateral laminars and facet joints, followed by an insertion of a blunt awl into the pedicle of the upper and lower vertebral body. The awl was adjusted to an ideal position under C-arm image, and then implantation of the pedicle-rod-screw system was performed. C-arm images were used to confirm the restoration of the normal height of vertebral body and sequence of spine. Medial facetectomy and laminectomy were performed at the affected vertebral level to find and remove the osseous and disc fragments that intruded into the anterior aspect of the dural sac. If dural laceration and CEH were found during operation, cauda equina fibers were released and replaced intradurally after enlargement of the dural defect. Duroplasty was performed to close or reconstruct dura mater using autologus patch of posterior layer of thoracolumbar fascia from the operative site. Additionally, posterolateral bony fusion was performed.

#### 2.2.2 Anterior approach

The indications for an anterior approach were as the follows. Firstly, patients were neurologically intact and younger than 40 years old, with more than 50% spinal canal encroachment by bony fragments and severe compression (more than 75%) and comminution. Secondly, there was an incomplete neurological lesion (cord, cauda equina, or root dysfunction) resulting from an anterior disc or bone. During the surgery, a left/right thoracoabdominal approach was used under general anesthesia in the lateral decubitus position. An incision was made in parallel to rib surface with a 2-rib level above the fractured vertebrae. The corpectomy and anterior decompression of the dural sac were performed. Within the space, a suitable cage was placed to collect morselized autogenous bone graft from the fractured vertebral body and rib resection. Retropulsed bony fragments were removed meticulously using angled curettes for decompression. Sharp surgical tools such as Kerisson rongeurs and power burrs were avoided in order to prevent from possible accidental iatrogenic cauda equina rootlets injury. Anterolateral fixation was used above and below the fractured vertebrae.

## 3 Results

The operation was completed successfully in 416 patients. There were no surgical complications in patients such as wound infection, dural laceration, or implantation failure. The CEH was observed in 12% (49 out of 416) patients. All of the herniation were identified in lumbar burst fracture. These patients had a mean age of 33.7 years old (range, 15–58 years old), with 40 males and 9 females. The mean follow-up was 28 months, with a range of 10 to 49 months. All the patients achieved satisfactory clinical outcomes at the final follow-up. The posterior approach group included 301 patients (n = 215 males and 86 females), with a CEH incident rate of 13% (40 out of 301, n = 35 males and 5 females). The anterior approach group included 115 patients (n = 80 males and 35 females) and the incident rate of CEH was 8% (9 out of 115, n = 5 males and 4 females). On their initial physical examinations, 44 patients (90%) with CEH had neurological deficits, including motor weakness, sensory dysesthesia, and hyporeflexia below the level of the fracture; the neurological injuries were asymmetric in most patients. Altered bladder and bowel function along with perianal anesthesia was found in 9 patients. Other 5 CEH patients (10%) showed neurologically intact. However, there were entrapments of a significant proportion of their cauda equina rootlets in the dorsal lamina fracture (Table 1), which were found during surgery.

**Table 1:**
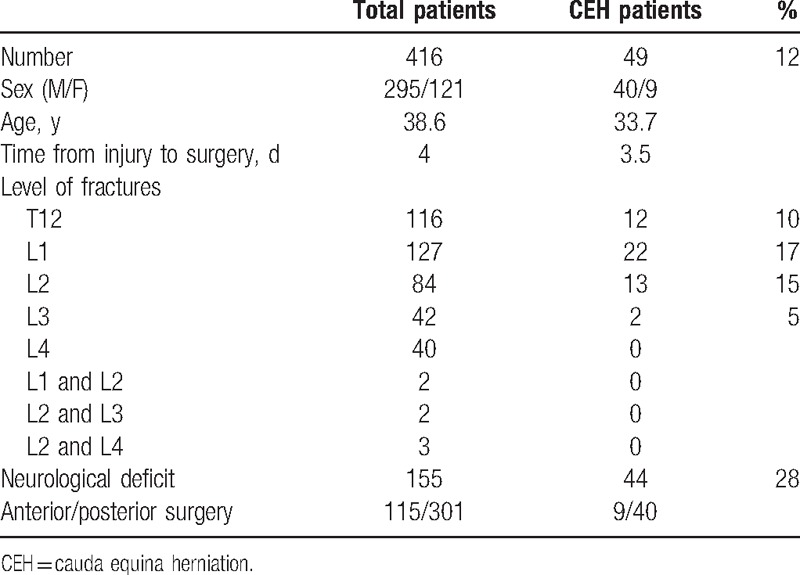
Summary of patient's demographic data.

**Figure 1:**
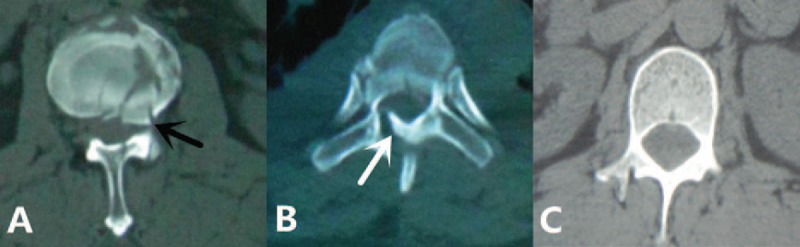
Different types of burst fractures in computed tomography scan: (A) asymmetric bone fragment intrusion into the spinal canal (the black arrow); (B) vertical lamina fracture (the white arrow); (C) normal vertebrae.

In those 9 patients undergoing anterior surgery, asymmetry of fracture fragment with intrusion into the spinal canal presented a sharp bone edge which might be the explanation on causing the dural laceration and CEH. Those without CEH showed more symmetrical fragment intrusion without sharp bony surfaces. In the posterior approach group, 47%patients (140 out of 301) had both vertebrae burst fractures and lamina fracture. The vertical lamina fracture could be seen on both anteroposterior plain radiographs and computed tomography scans (Fig. 1). The fracture could be a “greenstick” type, which disrupted the ventral cortex of the lamina only. Both vertebrae burst fractures and lamina fracture were observed in 95% patients with posterior CEH (38 out of 40).

**Figure 2:**
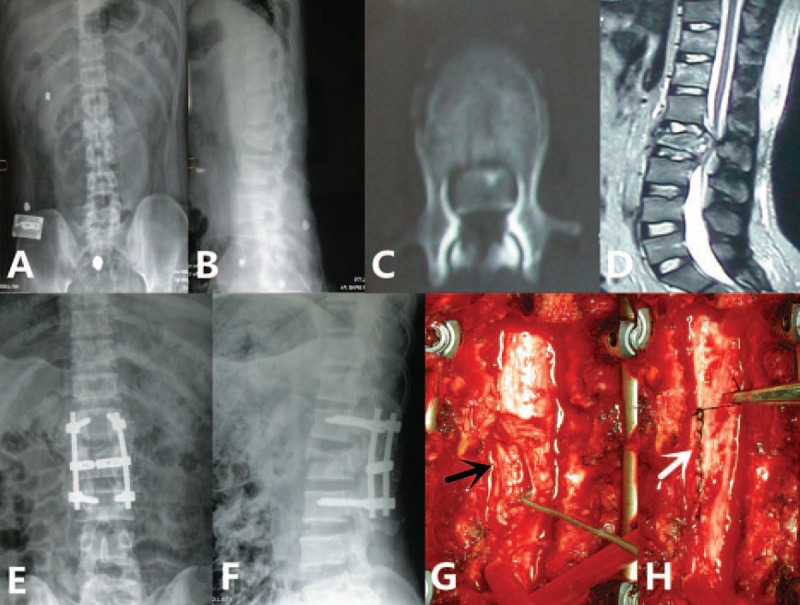
A 32-year-old male patient with L2 burst fracture, with ASIA C neurological impairment. (A and B) preoperative anteroposterior and lateral radiographs showed a L2 burst fracture; (C and D) computed tomography scan and magnetic resonance imaging showed bone fragment intrusion and severe spinal cord compression; (E and F) 3d postoperative anteroposterior and lateral radiographs showed vertebral height was restored, spinal cord was decompressed, and angular deformity was corrected by using posterior pedicle screw fixation; (G and H) intraoperative photograph showed dural laceration, herniated cauda equina rootlets (the black arrow) and the sutured dural sac (the white arrow).

During the operation, a few prolapsed, tangled cauda equina rootlets were found to be herniated through a small dural laceration. The tangled cauda equina rootlets were naked and devoid of arachnoid membrane cover. Some were with cerebrospinal fluid (CSF) leakage. The dural laceration was either dorsal or ventral with a longitudinal orientation. After restoration of the herniated cauda equina rootlets into the dural sac, the dural laceration was sealed with nonabsorbable sutures or reconstructed with autologus patch (Fig. 2). No patient showed neurological deteriorations following the dural repair or postoperative chronic pain syndrome. A continuous CSF leakage developed in 2 patients post primary repair of the dural laceration. This CSF leakage was successfully terminated with prolonged dressing.

## 4 Discussion

Vertical compressive and flexion forces are the general mechanism of injury for burst fractures of the thoracolumbar and lumbar spine. The bony end plates and cortical margins of the vertebral body can be fractured by axial loading due to high-energy forces,^[7–10]^ A radial displacement of corticocancellous bone fragments will occur when the vertebral body is compressed. Retropulsion of these bone and disc fragments into the spinal canal will impact the neural structures, resulting in displacement of the dural sac in the posterior direction and narrowness of the spinal canal. The anterior laceration of dural sac can be caused by the retropulsion of bone fragments; while the posterior displacement of dural sac can also cause laceration by the impalement on the sharp edges of the lamina fracture. Consequently, cauda equina rootlets extrude from the dural lacerated sites and a herniation forms. This is the typical pathological characteristic of the “traumatic CEH,” a result of thoracolumbar and lumbar burst fractures.

There are some types of neurological deficits in CEH patients, including motor weakness, sensory dysesthesia, and hyporeflexia below the fractured level. During operation, a few prolapsed, tangled cauda equina rootlets can be usually observed in the herniation through a small dural laceration. The vertical lamina fracture or retropulsed bone fragments can compress or entrap the extruded nerve rootlets and result in neurological deficit; while the remainder of rootlets can be decompressed simultaneously. Therefore, the neurological injuries are usually asymmetric in most patients. Some patients also show altered bladder and bowel function along with perianal anesthesia.

According to the intraoperative findings, there are 3 pathological types of the traumatic CEH: the ventral type showing numbness and muscle weakness in both legs with bowel or bladder dysfunction, which are caused by the retropulsed bone fragments; the lateral type showing asymmetric numbness and muscle weakness in one leg with rare bowel or bladder dysfunction, which are mainly caused by the free bone fragments or facet fracture; and the dorsal type showing similar symptoms as the ventral type, which are often caused by the sharp edges of the lamina fracture. In the present study, all of the herniation were identified in lumbar burst fractures. The possible reason is that the kyphotic spinal orientation helps to keep the neural elements remained in the anterior portion of the canal and within the dural sac following a fracture at the thoracolumbar junction. However, in the lower lumbar spine, the lordotic spinal alignment facilitates posterior extrusion of the cauda equina rootlets from the anteriorly tethered dural sac.

Some previous studies have reported the incident rates of posterior dural lacerations caused by thoracolumbar and lumbar fractures. For example, Camisa et al reported an 18% incident rate of posterior dural injuries in 60 patients with surgically treated burst fractures. All patients with dural injuries had neurological deficits.^[11]^ Keenen et al^[12]^ and Carl et al^[13]^ reported a 7.7% and 10% incidence of posterior dural tears in surgically treated patients, respectively.^[12,13]^ The incidence of CEH in the present study was 12%. However, this type of injury had not been reported in the orthopedics literature—particularly those series with a large number of patients. The possible reasons include as follows. Firstly, in orthopedics treatment, reduction and stabilization of the fracture are prioritized, while further destabilization of the fractured spine by laminectomy discourages an attempt for restoration of neural elements. Secondly, fractures are more commonly treated using a posterior approach. Incomplete decompression of fracture fragments may also cause the miss of anterior dural injuries. Finally, difficulty in control of bone and epidural bleeding may hinder such a herniation to be identified and fixed.

In operation, cauda equina rootlets were released and replaced intradurally after enlargement of the dural defect. The dura was sutured or reconstructed with autologus patch.^[14,15]^ As compared to posterior lacerations, anterior dural tears are more difficult to be repaired due to the smaller and deeper operative field at the site of decompression. Furthermore, the dural laceration may be linked with the pleural space if the injury occurs at L1 or above, which increases the risk of postoperative CSF leakage.^[16]^ Many investigators have found that burst fractures with an associated laminar fracture show a 100% sensitivity and 74% specificity for the presence of a dural tear.^[17–20]^ In the present study, both vertebrae burst fractures and lamina fracture were observed in 95% patients with posterior CEH. Therefore, patients with neurological deficits and a lumbar burst fracture in combination with a laminar fracture have an increased risk of a dural laceration and the possibility of traumatic CEH.

There are several limitations in this study. Firstly, the number of patients enrolled was small. Secondly, risk factors of traumatic CEH have not been included. Finally, the predictable magnetic resonance imaging findings about traumatic CEH have not been investigated in this study.

## 5 Conclusions

We found that the incident rate of traumatic CEH in the patients with thoracolumbar and lumbar burst fractures was about 12%. Reposition of cauda equina rootlets and repair of dural laceration were performed and rendered satisfactory clinical outcomes. Patients with neurological deficits and a laminar fracture in combination with a lumbar burst fracture have an increased risk of traumatic CEH, which should be recognized and considered in decision-making regarding operative treatment.
